# Prognostic factors for pediatric sudden sensorineural hearing loss: a systematic review and meta-analysis

**DOI:** 10.3389/fped.2026.1808182

**Published:** 2026-04-29

**Authors:** Zongyi Wu, Peili Zhang, Jianjun Sun, Mingfang Diao

**Affiliations:** 1 Department of Endoscopic Ear Surgery, Senior Department of Otorhinolaryngology Head and Neck Surgery, The Sixth Medical Center of PLA General Hospital of Beijing, Beijing, China; 2 Department of Otorhinolaryngology Head and Neck Surgery, Peking University International Hospital, Beijing, China

**Keywords:** children, meta-analysis, NLR (neutrophil-to-lymphocyte ratio), prognostic factors, sudden sensorineural hearing loss

## Abstract

**Objective:**

This systematic review and meta-analysis aimed to identify prognostic factors influencing hearing recovery in children with sudden sensorineural hearing loss (SSNHL), thereby providing a reference for developing prevention strategies and optimizing therapeutic interventions.

**Methods:**

We conducted a comprehensive search of PubMed, Web of Science, Cochrane Library, Embase, CNKI, and SinoMed databases to identify relevant studies published prior to 2024. Inclusion criteria encompassed pediatric patients with SSNHL (<18 years old) who had clearly documented audiological outcomes. We extracted data on demographic characteristics, clinical features, and treatment regimens to investigate the association between prognostic factors and hearing recovery.

**Results:**

This meta-analysis included 1,006 patients from 13 studies. The combined rates for partial and complete hearing recovery were 19.6% [95% confidence interval (CI): 13.6–25.7%, ***I²*** = 80.63%] and 18.6% (95% CI: 12.2–25.1%, ***I²*** = 85.99%), respectively. Better prognosis was associated with unilateral hearing loss [odds ratio (OR): 2.741, *P* = 0.004], age >12 years (OR: 1.911, *P* = 0.034), treatment delay ≤ 14 days (OR: 6.402, *P* < 0.001), and an ascending audiogram curve (OR: 6.910, *P* < 0.001). Conversely, poorer outcomes were associated with profound hearing loss curves (OR: 0.376, *P* < 0.001) and initial pure-tone average (PTA) thresholds >80 dB HL (OR: 0.451, *P* = 0.005).

**Conclusion:**

Early intervention (≤14 days) and targeted treatment for profound hearing loss (PTA > 80 dB HL) hold promise for improving pediatric outcomes. Systemic inflammatory markers (neutrophil-to-lymphocyte ratio) and age-specific pathophysiological mechanisms warrant further investigation. Conducting multicenter prospective studies is essential to validate these findings and establish standardized diagnostic and treatment protocols for pediatric SSNHL.

**Systematic Review Registration:**

https://www.crd.york.ac.uk/PROSPERO/view/CRD42024612409, CRD42024612409.

## Introduction

The clinical diagnostic criteria for sudden sensorineural hearing loss (SSNHL) vary slightly across different national guidelines, summarized as follows: (1) The German guidelines define SSNHL as “sudden onset (typically unilateral) sensorineural hearing loss,” while the American and Chinese guidelines specify “sudden sensorineural hearing loss occurring within 3 days.” (2) According to the Chinese guidelines, hearing loss is defined as ≥20 dB HL in at least two adjacent frequencies, while the American guidelines define it as ≥30 dB affecting at least three consecutive frequencies. SSNHL is predominantly unilateral, although bilateral cases may occur simultaneously or sequentially, as noted in German, American, and Chinese guidelines. (3) All three guidelines agree that SSNHL has no identifiable systemic or local etiology. (4) SSNHL may be accompanied by tinnitus, ear fullness, vertigo, nausea, or vomiting (German, American, and Chinese guidelines). (5) The German guidelines also note that hyperacusis, hyperesthesia, and percutaneous sensory abnormalities may be present (39). According to the United Nations Convention on the Rights of the Child, a child refers to any person under the age of 18. Therefore, SSNHL in children refers to sudden hearing loss occurring in patients aged 0–18 years (2). In the Aachen region of Germany, among a population of approximately 250,000, the incidence rate of idiopathic SSNHL in children is one in 10,000. This rate is only one-tenth to one-twentieth of the incidence rate observed among adults during the same period, which is significantly lower than that observed in adults (3). According to data from the U.S. Medical and Pharmaceutical Claims Database, those under 18 years account for 6.6% of SSNHL cases, those under 14 years account for 3.5%, and those under 9 years account for only 1.2% (4). This is a rare but serious condition that may adversely affect the social adaptation, behavior, and psychological development of children during their formative years. At present, no specific epidemiological studies exist for SSNHL in the pediatric population. Despite the significant impact of this disorder, its rarity and the limitations of existing research have resulted in an insufficient description of prognostic factors for SSNHL in children.

While the precise etiology remains elusive, viral infections, problems with blood flow in the inner ear, and immune system issues are considered predominant causes of pediatric SSNHL based on existing evidence (5, 6). Systemic glucocorticoids are predominant, with intratympanic steroid injection (ITS) used as salvage therapy (7). Bilateral SSNHL is more often associated with suspected etiologies, such as inner ear malformations and viral infections (8).

Due to the low incidence of SSNHL in children, it is challenging to conduct prospective clinical studies to identify its prognostic factors. In addition, most studies investigating prognostic factors for SSNHL are retrospective in nature, characterized by small sample sizes and varying definitions of hearing recovery. Previous meta-analyses have been conducted on SSNHL in children, but the included studies were limited to English-language literature, and some research was outdated (9). Therefore, we conducted a systematic review and meta-analysis specifically focused on prognostic factors for SSNHL in children. This study included relevant literature published in Chinese and English journals, aiming to systematically review related research and identify factors significantly associated with hearing recovery in children with SSNHL.

## Materials and methods

### Search strategy

**Figure 1:**
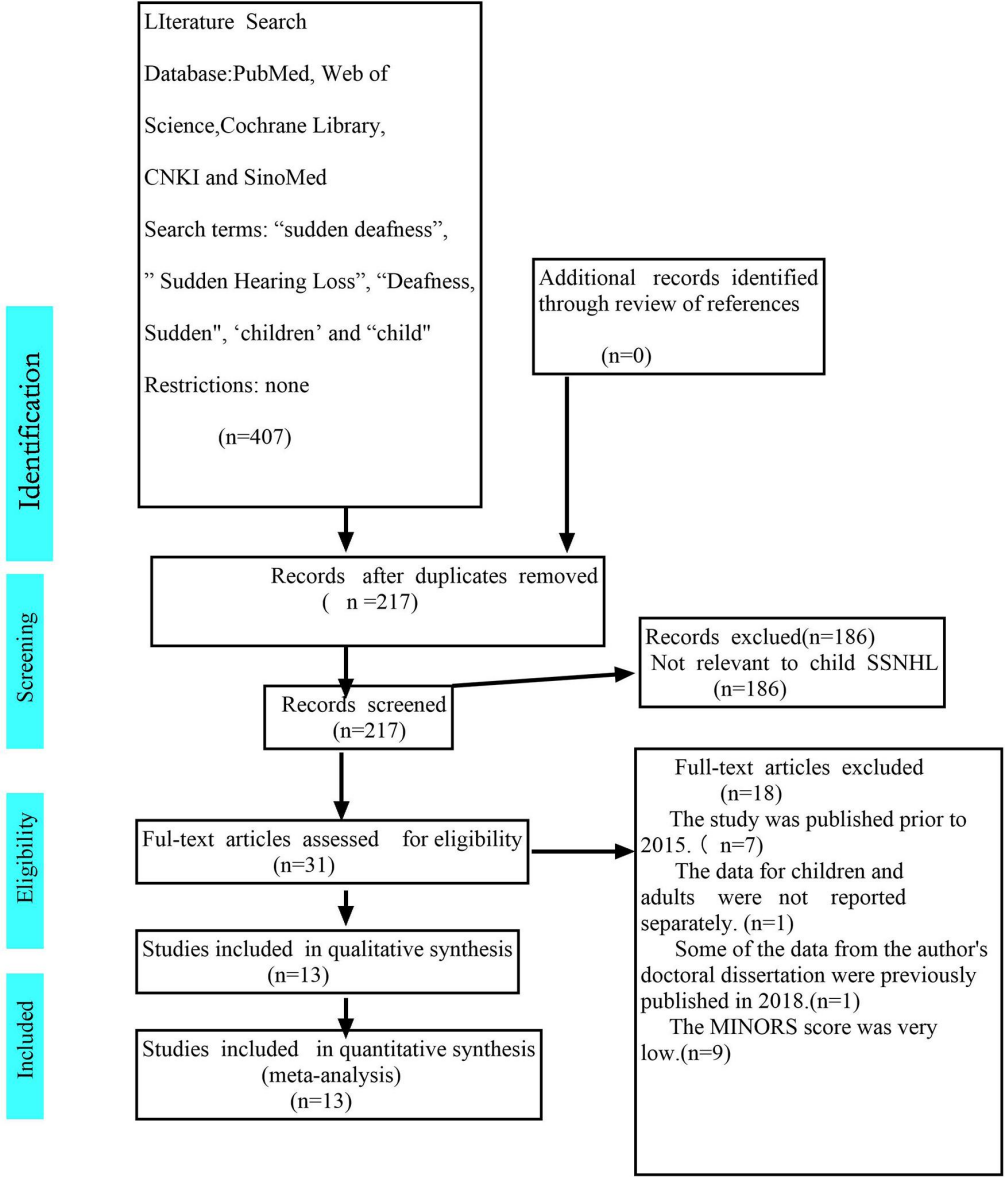
PRISMA flow diagram of literature screening and inclusion process.

We conducted a systematic search of articles published prior to 2024 in PubMed, Web of Science, Cochrane Library, Embase, CNKI, and SinoMed databases, and screened the reference lists of relevant articles. The included studies were case series and cohort studies focusing on SSNHL in patients under 18 years of age. Search strategies were developed for each database incorporating keywords and Medical Subject Headings (MeSH) terms related to “sudden deafness,” “sudden sensorineural Hearing Loss,” “deafness, sudden,” “children,” and “child”. The detailed PubMed search strategy is provided in Figure 1. Two reviewers (ZW and PZ) conducted an initial assessment of eligible studies through a double-blind independent review process. Any discrepancies were resolved through discussion or consultation with a third reviewer (JS or MD).

### Data collection

Inclusion criteria: Articles were required to meet all of the following conditions:

1. Document audiological characteristics;
2. Report clinical outcomes in pediatric and adolescent populations (0–18 years);
3. Focus on patients diagnosed with SSNHL;
4. Be peer-reviewed and published in 2015 or later (the year the Chinese Society of Otolaryngology-Head and Neck Surgery revised its clinical guidelines for SSNHL).

Exclusion criteria: Studies were excluded if they met any of the following considerations:

1. Non-research publications, including case reports, reviews, and editorial commentaries;
2. Inadequate methodological quality according to the MINORS criteria;
3. Failure to provide stratified data analysis for pediatric and adult populations;
4. Conference abstracts, unpublished data, and non-peer-reviewed materials.

Conflict resolution and screening process:

1. Any disagreements in study selection were resolved through consensus discussion.
2. The reference lists of included articles underwent manual cross-checking.
3. Only full-text, peer-reviewed journal articles were ultimately considered for inclusion.

The systematic review extracted the following variables from eligible studies:

1. Study methodology
  - Publication details (year, country);
  - Study design (prospective/retrospective, controlled/uncontrolled);
  - Inclusion and exclusion criteria;
  - Outcome classification system (hearing recovery grades: no recovery, partial recovery, complete recovery);
  - Sample size (number of participants and/or affected ears).
2. Demographic characteristics
  - Gender distribution;
  - Age at diagnosis.
3. Audiological parameters
  - Unilateral vs. bilateral involvement;
  - Baseline hearing thresholds (pretreatment);
  - Associated symptoms (tinnitus, vertigo, ear fullness);
  - Audiogram configuration (specific frequency hearing patterns).
4. Treatment characteristics
  - Use of systemic corticosteroids, intratympanic injections, or combination therapy;
  - Time interval from symptom onset to treatment initiation.

### Outcomes assessment

The primary outcomes were categorized as no recovery, partial recovery, or complete recovery. The methodological quality (risk of bias) of included observational studies was assessed using the Methodological Index for Non-Randomized Studies (MINORS) instrument (11). Following the approach described in a previous study (12), we categorized quality as follows: non-comparative studies—very low (0–4), low (5–8), moderate (9–12), and high (13–16); comparative studies—very low (0–6), low (7–12), moderate (13–18), and high (19–24) This study followed the Preferred Reporting Items for Systematic Reviews and Meta-Analyses (PRISMA) guidelines (13).

### Statistical analysis

We conducted a meta-analysis using the SPSS AU online platform. For categorical variables, pooled rates with 95% confidence intervals (CI) were reported. For continuous variables, means with 95% confidence intervals were calculated. Heterogeneity was assessed using the ***I*²** statistic. When ***I*²** = 0, a fixed-effects model was applied; when ***I*²** > 0, a random-effects model was employed (10).

## Results

### Study characteristics

Figure 1 (PRISMA flow diagram) illustrates the literature screening process, with a total of 13 studies meeting the inclusion criteria. One study, a doctoral dissertation, was excluded because part of its data had already been published in 2018. Another study was excluded for failing to adequately stratify data between children and adults. Of the included studies, nine were case series (rated as Level 4 evidence according to the Oxford Centre for Evidence-Based Medicine grading system) and four studies were cohort studies (rated as Level 3 evidence according to the Oxford Centre for Evidence-Based Medicine grading system). The MINORS scores for the included studies are presented in Table 1. The ideal MINORS score is 16 points for case series and 24 points for cohort studies; however, none of the included studies achieved the maximum score. These 13 included studies collectively enrolled 1,022 patients. However, three studies did not report the number of ears with hearing loss, making it impossible to calculate the exact total number of ears included. Five studies explicitly specified the duration of follow-up.

**Table 1:** Characteristics of included studies.

Author and year	Country	Study type	Patients	Total ears analyzed	Follow-up ears	Follow-up (months)	No recovery (ears), *n* (%)	Partial recovery (ears), *n* (%)	Complete recovery (ears), *n* (%)	MINORS score
Xu and Fu, 2024 (17)	China	Case series	109	NR	NR	NR	51 (51.4)	43 (39.4)	10 (9.2)	6
Xiao et al., 2023 (21)	China	Case series	251	317	317	NR	222 (70.03)	54 (17.04)	41 (12.93)	9
Lu et al., 2019 (26)	China	Cohort	25	26	26	NR	9 (34.6)	10 (38.4)	7 (26.9)	13
Ha et al., 2019	South Korea	Cohort	42	NR	NR	NR	24 (57.1)	11 (26.2)	7 (16.7)	17
Qian et al., 2018 (16)	China	Case series	75	78	78	NR	22 (29.3)	21 (28.0)	32 (42.7)	14
Kim et al., 2018 (20)	South Korea	Case series	67	67	67	2.5 (1–3)	30 (44.8)	13 (19.4)	24 (35.8)	13
Chen et al., 2018 (8)	China	Case series	101	129	129	7.4 (4–12)	95 (73.6)	13 (10.1)	21 (16.3)	10
Li et al., 2016 (22)	China	Case series	136	151	151	NR	94 (62.2)	43 (28.5)	14 (9.3)	7
Kizilay and Koca Ç, 2016 (23)	Turkey	Case series	14	16	16	6.8 (1–20)	13 (81.25)	0 (0.00)	3 (18.75)	7
Dedhia and Chi, 2016 (1)	USA	Case series	6	6	6	27 (1–83)	NR	NR	0 (0.0)	6
Li et al., 2015 (15)	China	Case series	101	113	113	0.61 (1–6)	72 (63.7)	30 (26.6)	11 (9.7)	8
Chung et al., 2015 (18)	South Korea	Cohort	37	37	37	NR	10 (27.0)	9 (24.3)	18 (48.6)	11
Ao et al., 2015 (14)	China	Cohort	44	44	44	NR	17 (38.6)	9 (20.4)	14 (31.8)	11

MINORS, methodological index for non-randomized studies; NR, not reported.

The definitions of hearing recovery adopted in the included studies are summarized in Table 2. Among these, four studies used the criteria from the Chinese Medical Association's Guidelines for the Diagnosis and Treatment of SSNHL, defining hearing recovery as follows: no recovery (hearing improvement <15 dB HL), partial recovery (hearing improvement >15 dB HL), and complete recovery (return to normal or pretreatment levels) (14–17). Six studies defined hearing recovery using Siegel criteria as follows: no recovery [pure-tone average (PTA) > 45 dB HL], partial recovery (PTA between 25 and 45 dB HL with ≥15 dB improvement), and complete recovery (PTA < 25 dB HL) (8, 18–21). One study defined hearing recovery as follows: no recovery (hearing improvement <15 dB HL), partial recovery (hearing improvement >15 dB HL), or complete recovery (PTA<25 dB HL) (22). Another study categorized hearing recovery as follows: no recovery (no change), partial recovery (undefined), or complete recovery (restoration to the level of hearing in the unaffected ear) (23). One of the studies did not report specific details on the definition of hearing recovery (1).

**Table 2:** Definitions of hearing recovery criteria used in included studies.

Author and year	No recovery	Partial recovery	Complete recovery
Xu and Fu, 2024 (17)	<15 dB gain	>15 gain	Normal/pretreatment level
Xiao et al., 2023 (21)	PTA > 45 dB HL	PTA 25–45 dB HL with ≥15 dB gain	PTA < 25 dB HL
Lu et al., 2019 (26)	PTA > 45 dB HL	PTA 25–45 dB HL with ≥15 dB gain	PTA < 25 dB HL
Ha et al., 2019 (19)	PTA > 45 dB HL	PTA 25–45 dB HL with ≥15 dB gain	PTA < 25 dB HL
Qian et al., 2018 (16)	<15 dB gain	>15 dB gain	Normal/pretreatment level
Kim et al., 2018 (20)	PTA > 45 dB HL	PTA 25–45 dB HL with ≥ 15 dB gain	PTA < 25 dB HL
Chen et al., 2018 (8)	PTA > 45 dB HL	PTA 25–45 dB HL with ≥15 dB gain	PTA < 25 dB HL
Li et al., 2016 (22)	<15 dB gain	>15 dB gain	PTA < 25 dB HL
Kizilay and Koca Ç, 2016 (23)	No change	Not reported	Restore unaffected ear level
Dedhia and Chi, 2016 (1)	Not reported	Not reported	Not reported
Li et al., 2015 (15)	<15 dB gain	>15 dB gain	Normal/pretreatment level
Chung et al., 2015 (18)	PTA > 45 dB HL	PTA 25–45 dB HL with ≥15 dB gain	PTA <25 dB HL
Ao et al., 2015 (14)	<15 dB gain	>15 dB gain	Normal/pretreatment level

A total of 217 unique records were identified for this study. After removing duplicates and screening titles and abstracts, 31 studies were selected for full-text review. Based on predefined inclusion criteria, 18 studies were excluded, including seven studies published before 2015 and one study that did not report separate data for children and adults (24). One doctoral dissertation containing previously published data and nine studies with suboptimal MINORS were also excluded (25).

### Demographic

We conducted a meta-analysis of the basic demographic characteristics of SSNHL in children. We analyzed two studies measuring gender distribution by number of affected ears and 11 case-based studies, revealing that 54.3% of hearing loss cases occurred in boys (95% CI: 50.8%–57.8%, ***I*²** = 0.00%). Regarding age characteristics, a pooled analysis of four studies categorizing age into ≤12 years versus 13–18 years estimated that 38.2% belonged to the younger age subgroup (95% CI: 8.8%–67.5%, ***I*²** = 96.20%), while 61.8% were in the older subgroup (95% CI: 32.5%–91.2%, ***I*²** = 96.20%). A continuous variable meta-analysis of five studies yielded an overall mean age of 13.46 years (95% CI: 12.47–14.45, ***I*²** = 70.95%). Lateralization analysis from 12 studies indicated unilateral hearing loss in 85.2% of cases (95% CI: 78.2%–92.3%, ***I*²** = 94.57%) (Table 3).

**Table 3:** Characteristics of pediatric SSNHL.

Factor	No. studies	Meta-analyzed prevalence or mean	95% CI	*I*^2^ (%)	Unit of analysis
Male	11	0.543	0.508, 0.578	0.000	Per patient
Female	11	0.457	0.422, 0.492	0.000	Per patient
Age, year					
≤12	4	0.382	0.088, 0.675	96.20	Per patient
13–18	4	0.618	0.325, 0.912	96.20	Per patient
Unilateral	12	0.852	0.782, 0.923	94.57	Per ear
Initial PTA, dB HL					
<40	4	0.111	0.052, 0.170	78.98	Per ear
41–60	4	0.136	0.052, 0.219	88.64	Per ear
61–80	4	0.198	0.140, 0.256	61.11	Per ear
>80	4	0.545	0.453, 0.638	76.88	Per ear
Audiogram					
Ascending	4	0.168	0.056, 0.280	82.43	Per patient
Descending	4	0.16	0.081, 0.239	62.92	Per patient
Concave/convex	2	None	None	None	
Flat	4	0.266	0.158, 0.374	72.59	Per patient
Profound	4	0.285	0.190, 0.380	61.29	Per patient
Additional symptoms					
Tinnitus	8	0.567	0.407, 0.727	93.16	Per patient
Vertigo	8	0.218	0.131, 0.306	81.94	Per patient
Fullness	4	0.310	0.118, 0.502	88.83	Per patient
Systemic steroid	7	0.949	0.906, 0.992	92.08	Per patient
ITS	4	0.285	0.141, 0.429	82.75	Per patient
Time to treatment, days					
≤7	3	0.789	0.736, 0.842	0.000	Per patient
Partial improvement	10	0.196	0.136, 0.257	80.63	Per ear
Total improvement	10	0.186	0.122∼0.251	85.99	Per ear
Continuous variables					
Age, year	5	13.46	12.470, 14.451	70.95	Per patient
Initial PTA, dB HL	3	72.155	59.324, 84.987	83.73	Per patient
Treatment delay, days	3	5.752	3.500∼8.003	74.45	Per patient

### Audiological characteristics

We conducted a meta-analysis to evaluate the initial degree of hearing loss, baseline hearing thresholds, configuration of audiometric curves, and associated symptoms. Hearing loss severity was categorized into four levels based on the initial pure-tone average threshold (measured in decibels): <40, 41–60, 61–80, and >80 dB HL. This meta-analysis included four studies. The prevalence of comorbidities among patients with baseline PTA <40 dB HL was 11.1% (95% CI: 5.2%–17.0%; ***I*²** = 78.98%), while 13.6% (95% CI: 5.2%–21.9%; ***I*²** = 88.64%) had an initial PTA of 41–60 dB HL. Moreover, 19.8% of patients with an initial PTA of 61–80 dB HL (95% CI: 14.0%–25.6%; ***I*²** = 61.11%) had this condition, while the highest prevalence was observed in patients with an initial PTA >80 dB HL, reaching 54.5% (95% CI: 45.3%–63.8%) (Table 3).

Three studies reported baseline hearing thresholds expressed as mean and standard deviation. The pooled mean initial hearing threshold across these studies was 72.155 dB HL (95% CI: 59.324–84.987 dB; ***I*²** = 83.73%). Hearing curve configurations were classified into five types: ascending, descending, concave/convex, flat, and profound. We conducted a meta-analysis reporting case numbers for each type. Due to insufficient data, the concave/convex type was excluded from analysis, as only two studies described this configuration. For the remaining four curve types, data from four studies were included in this analysis. The pooled prevalence of comorbidity among patients with ascending curves was 16.8% (95% CI: 5.6%–28.0%; ***I*²** = 82.43%), while that for descending curves was 16.0% (95% CI: 8.1%–23.9%; ***I*²** = 62.92%). Flat hearing curves were observed in 26.6% of patients (95% CI: 15.8%–37.4%; ***I*²** = 72.59%), while complete deafness curves were present in 28.5% (95% CI: 19.0%–38.0%; ***I*²** = 61.29%) (Table 3).

### Symptoms

Eight studies reported the prevalence of tinnitus, with a pooled prevalence of 56.7% (95% CI: 40.7%–72.7%; ***I*²** = 93.16%). Similarly, eight studies documented vertigo, yielding a pooled prevalence of 21.8% (95% CI: 13.1%–30.6%; ***I*²** = 81.94%). Four studies reported the prevalence of ear fullness, with a pooled prevalence of 31.0% (95% CI: 11.8%–50.2%; ***I*²** = 88.83%) (Table 3).

### Treatment

When classifying the time to initial diagnosis using categorical variables, studies varied in their categorization of time intervals and measurement units. Only the ≤7-day interval had sufficient data (*n* = 3) for meta-analysis. The proportion of patients starting treatment within ≤7 days after symptom onset was 78.9% (95% CI: 73.6%–84.2%; ***I*²** = 0%). For studies reporting initial diagnosis time as a continuous variable, three met the criteria for meta-analysis. The pooled mean initial diagnosis time was 5.752 days (95% CI: 3.500–8.003 days; ***I*²** = 74.45%). Seven studies indicated that 94.9% of patients received systemic steroid therapy (95% CI: 90.6%–99.2%, ***I*²** = 92.08%). Four studies reported that 28.5% of patients underwent intratympanic injection therapy (95% CI: 14.1%–42.9%, ***I*²** = 82.75%). This study included 920 ears from 10 studies. Partial hearing recovery was achieved in 19.6% of ears (95% CI: 13.6%–25.7%, ***I*²** = 80.63%) and complete hearing recovery in 18.6% of ears (95% CI: 12.2%–25.1%, ***I*²** = 85.99%) (Table 3).

### Factors related to hearing prognosis

Meta-analysis identified the following factors associated with hearing recovery (partial or complete): Unilateral hearing loss (odds ratio: 2.741; 95% confidence interval: 1.370–5.483) was associated with a better prognosis. Age between 13 and 18 years (odds ratio: 1.911; 95% confidence interval: 1.051–3.475) and older age (mean difference: 0.568; 95% confidence interval: 0.188–0.948) were associated with better outcomes. Treatment delay within 14 days (odds ratio: 6.402; 95% confidence interval: 2.940–13.940) was associated with favorable prognosis. Lower initial pure-tone average hearing thresholds (mean difference: −0.875; 95% confidence interval: −1.170 to −0.029) were associated with better recovery. An ascending audiogram curve (odds ratio: 6.910; 95% confidence interval: 3.825–12.483) was associated with a favorable prognosis, whereas a profound hearing loss curve (odds ratio: 0.375; 95% confidence interval: 0.273–0.517) was associated with an unfavorable prognosis (Table 4).

**Table 4:** Meta-analysis of factors influencing hearing recovery.

Factor	No. of studies	Odds ratio or mean difference	95% CI	*P*	*I*^2^ (%)
Categorical variables					
Male	10	0.859	0.581, 1.270	0.446	37.37
Unilateral	8	2.741	1.370, 5.483	**0**.**004**	57.71
Age 13–18 years	3	1.911	1.051, 3.475	**0**.**034**	0.00
PTA >80 dB	7	0.517	0.296, 1.099	0.093	76.95
Audiogram					
Ascending	9	6.910	3.825, 12.483	**<0**.**001**	0.00
Descending	9	1.575	0.693, 3.579	0.278	60.00
Flat	9	1.150	0.835, 1.583	0.393	0.00
Profound	9	0.376	0.273, 0.517	**<0**.**001**	4.66
Concave/convex	2	0.717	0.208, 2.474	0.599	49.21
Additional symptoms					
Tinnitus	10	1.221	0.580, 2.572	0.581	74.30
Vertigo	10	0.997	0.696, 1.429	0.997	14.10
Fullness	5	1.143	0.653, 2.000	0.640	0.00
ITS	3	0.552	0.227, 1.343	0.190	40.20
Treatment delay ≤14 days	5	6.402	2.940, 13.940	**<0**.**001**	0.00
Continuous variables					
Age, year	3	0.568	0.188, 0.948	**0**.**003**	0.00
Treatment delay, day	3	−0.977	−0.977, 0.319	0.329	13.15
Initial PTA, dB HL	2	−0.875	−1.170,−0.029	**<0**.**001**	0.00

ITS, intratympanic steroid injection.

Bold indicates statistically significant.

### Sensitivity analyses

We performed sensitivity analysis on the initial meta-analysis results with ***I*²** > 50% and found that an initial PTA > 80 dB HL (OR: 0.450; 95% CI: 0.260–0.783) was associated with a poorer prognosis, while the remaining prognostic factors did not show statistical significance (Table 5).

**Table 5:** Sensitivity analyses of factors influencing hearing recovery.

Factor	Study	Odds ratio or mean difference	95% CI	*P*	*I*^2^ (%)
Categorical variables					
Unilateral	(-) Li et al. (22)	2.202	1.167, 4.700	**0**.**017**	45.33
PTA >80 dB	(-) Li et al. (22)	0.451	0.260, 0.783	**0**.**005**	60.08
Tinnitus	(-) Li et al. (15)	1.554	0.749, 3.223	0.236	68.41
Descending	(-) Xu and Fu (17)	0.619	0.570, 2.573	0.619	50.07
Continuous variables					
Treatment delay, days	(-) Kizilay and Koca (23)	0.520	−0.952, −0.089	**0**.**018**	13.15

Bold indicates statistically significant.

## Discussion

Due to the rarity of SSNHL in children, there is currently limited research on this condition. Unlike previous reviews (9), our study included not only English-language research but also relevant studies published in Chinese. Our updated meta-analysis included 13 studies, all published after 2015. This study enrolled 1,022 patients from 13 studies; 19.6% of ears achieved partial hearing recovery (95% CI: 13.6%–25.7%, ***I*²** = 80.63%), while 18.6% of ears achieved complete hearing recovery (95% CI: 12.2%–25.1%, ***I*²** = 85.99%). The substantial heterogeneity observed in pooled recovery rates (***I*²** > 80%) likely stemmed from inconsistent definitions of hearing recovery across studies (Table 1). This heterogeneity was further amplified by variations in treatment protocols, course durations, and dosage regimens.

Our study found that children aged 13–18 with SSNHL had a better prognosis than those aged ≤12 years. Older children with SSNHL generally had a relatively better prognosis. Younger children often experience delayed medical care due to their inability to clearly express the discomfort caused by hearing impairment, leading to missed opportunities for optimal treatment. Children with sudden sensorineural hearing loss who receive treatment within ≤14 days or even shorter delays demonstrated better outcomes. Current research indicates that a shorter interval between symptom onset and treatment is a significant positive prognostic factor. Early detection and prompt treatment should be advocated to achieve favorable outcomes.

Patients with unilateral hearing loss tended to have better hearing outcomes than those with bilateral hearing loss. Patients with bilateral SSNHL were younger, had a higher proportion of suspected etiologies, exhibited a greater rate of severe deafness, and demonstrated a higher incidence of tinnitus (8).

In this study, tinnitus was not associated with prognosis (*P* = 0.581, ***I*²** = 74.3%). This finding differs from previous reviews suggesting a positive correlation between tinnitus and prognosis, and our meta-analysis for this specific outcome revealed significant heterogeneity. Tinnitus is considered a positive prognostic factor, potentially reflecting residual neural activity that may aid auditory recovery, though its role remains controversial. Some studies suggest that patients with tinnitus experience better hearing recovery compared to those without tinnitus (9, 16, 20, 21), while others report poorer hearing prognosis in tinnitus-afflicted individuals (29).

Consistent with findings from a prior review (9), we found that vertigo was not associated with the prognosis of pediatric SSNHL, although many scholars believe that it is. A meta-analysis found that the presence of acute vestibular syndrome and abnormal vestibular function test results were associated with poor hearing recovery (35). Patients with concomitant vestibular dysfunction often exhibit poor prognosis. Given the close proximity of the vestibular system and cochlea, both structures are supplied by the same artery. When blood supply to this region is compromised, sudden hearing loss accompanied by vertigo may occur, suggesting that inner ear damage may involve the vestibular system (36). Research has revealed that vestibular end-organ dysfunction manifests in diverse patterns that do not strictly correspond to neurovascular distribution areas. Vestibular impairment is not solely attributable to microcirculatory disorders but may result from other causes, including viral infections and immune-mediated diseases (37). At present, there is limited research on the impact of vestibular function on the prognosis of children with sudden sensorineural hearing loss. Future studies could conduct comparative analyses to explore this topic in greater depth.

Our research indicates that patients with SSNHL exhibiting an ascending audiogram curve demonstrate a more favorable prognosis, potentially due to the underlying mechanism of endolymphatic hydrops, which is responsive to drug therapy (38). In contrast, children with SSNHL exhibiting a profound audiometric curve pattern have a poorer prognosis. This pattern indicates severe damage to the hair cells within the cochlea. Research has shown that recovery from damage to the cochlear apex is better than recovery from damage to the cochlear middle, which in turn is better than recovery from damage to the cochlear base. The causes of damage to the cochlear base differ from those affecting the cochlear apex. This may be due to differences in metabolic function between apical and basal hair cells, or differences in their blood supply (28).

The exact causes of SSNHL remain unclear, but it may be associated with viral infections, impaired blood flow to the inner ear, and autoimmune reactions (5). Among these, infections such as cytomegalovirus or the Epstein–Barr virus are believed to cause cochlear damage by directly damaging nerve cells or triggering an immune response. The neutrophil-to-lymphocyte ratio serves as an easily accessible prognostic marker for predicting the outcome of SSNHL in children (19). Bulğurcu et al. found that the neutrophil-to-lymphocyte ratio serves as an effective predictor of prognosis in pediatric sudden sensorineural hearing loss, demonstrating high accuracy in identifying outcomes. Their study also indicated that patients who did not recover after treatment exhibited significantly higher neutrophil-to-lymphocyte ratios (NLRs) (30). Research has demonstrated that NLR values show a significant positive correlation with pure-tone thresholds prior to treatment, indicating that NLR levels may reflect disease severity. Furthermore, children with sudden sensorineural hearing loss have exhibited significantly higher NLR values than healthy controls, suggesting that systemic inflammation may be involved in the pathogenesis of sudden sensorineural hearing loss in children (27). These findings collectively support the potential value of NLR as a prognostic biomarker for sudden sensorineural hearing loss in children, with elevated NLR levels indicating poorer outcomes (19). Elevated NLR levels correlate with severe hearing loss and poorer recovery, reinforcing systemic inflammation's role in pediatric SSNHL.

Unlike an earlier review (9), which only looked at studies in English, our review included Chinese studies and found that a higher percentage of patients (94.9% compared to 64.5%) received systemic steroids, indicating differences in treatment practices by region. Moreover, 28.5% of the patients in our review received ITS as salvage treatment, which is similar to the 30.8% reported in a previous review (9).Currently, there is no standardized treatment protocol for pediatric SSNHL. Our analysis did not reveal a statistically significant association between corticosteroid therapy (whether systemic or intratympanic) and hearing recovery outcomes. However, this finding must be interpreted with extreme caution due to several critical limitations. Only one study in the included literature formally analyzed this association. Systemic steroid use was nearly universal (94.9%) among patients in the included studies, precluding meaningful comparison with untreated controls. Furthermore, significant variations existed in steroid type, dosage, route of administration, and treatment duration, obscuring any potential therapeutic effect. Nevertheless, clinical guidelines for SSNHL in China, Germany, and the United States continue to recommend corticosteroids as first-line therapy. Furthermore, U.S. clinical practice guidelines recommend corticosteroids as initial treatment within 2 weeks of symptom onset, and recommend intratympanic steroid therapy when symptoms do not fully resolve between 2 and 6 weeks (31). Chinese clinical practice guidelines recommend corticosteroid therapy as initial treatment during the acute phase of SSNHL (within 3 weeks), with intratympanic steroids as a supplementary treatment (32). Similarly, German clinical practice guidelines for sudden sensorineural hearing loss also designate corticosteroid therapy as the first-line treatment, with a standard dose of 60 mg/day. The German guidelines recommend at least 250 mg/day for 3 days. High-dose therapy (500 mg/day for the first 3 days followed by 60 mg/day for 11 days) showed no significant advantage over standard-dose therapy (60 mg/day for 14 days). Although evidence is insufficient for a strong recommendation, systemic steroids remain the most widely used first-line treatment and can be considered the current standard of care (33). Research indicates that hormone therapy can suppress inflammatory responses, activate ion transport in the stria vascularis and the spiral ligament within the cochlear duct, regulate endolymphatic homeostasis, reduce endolymphatic hydrops, and enhance cochlear blood circulation (34). For patients with poor treatment responses, intratympanic or postauricular corticosteroid injections may serve as salvage therapy (6). The advantages of ITS include (1) reduced systemic adverse effects, (2) direct action on the inner ear, bypassing the blood–labyrinth barrier, and (3) higher drug concentration in the perilymph (25). This methodological variability precludes definitive conclusions regarding corticosteroid efficacy in the pediatric idiopathic sudden sensorineural hearing loss prognosis. Despite the widespread use of systemic steroids (94.9%), their efficacy remains inconclusive due to heterogeneous dosing and administration protocols. ITS, employed in 28.5% of cases, may offer localized therapeutic benefits but requires pediatric-specific validation.

This study has several limitations. The retrospective design of the included studies introduced selection bias, particularly in treatment allocation and outcome reporting. Furthermore, inconsistent definitions of hearing recovery across studies (e.g., Siegel criteria vs. Chinese guidelines) and high heterogeneity (*I*^2^ > 80% for recovery rates) may have confounded pooled estimates. Small sample sizes and variable treatment protocols further constrained inferences about causality.

## Conclusion

Early intervention (≤14 days) and intensified therapy for severe cases (PTA >80 dB) are recommended for treatment of SSNHL. NLR may serve as a cost-effective prognostic biomarker. Future research should prioritize multicenter randomized trials to standardize treatment protocols and elucidate pediatric-specific etiologies, such as viral or autoimmune mechanisms. Addressing these gaps will enhance evidence-based management and long-term outcomes for the vulnerable pediatric population.

## Data Availability

The original contributions presented in the study are included in the article/supplementary material, further inquiries can be directed to the corresponding authors.

## References

[1] Dedhia K Chi DH. Pediatric sudden sensorineural hearing loss: etiology, diagnosis and treatment in 20 children. Int J Pediatr Otorhinolaryngol. (2016) 88:208–12. 10.1016/j.ijporl.2016.07.003 27497416

[2] Hou Z. Research progress on clinical characteristics and prognosis of sudden deafness patients across different age groups. J Audio Speech Pathol. (2013) 21:418–21. 10.3969/j.issn.1006-7299.2013.04.029

[3] Chen YS Emmerling O Ilgner J Westhofen M. Idiopathic sudden sensorineural hearing loss in children. Int J Pediatr Otorhinolaryngol. (2005) 69:817–21. 10.1016/j.ijporl.2005.01.015 15885335

[4] Alexander TH Harris JP. Incidence of sudden sensorineural hearing loss. Otol Neurotol. (2013) 34:1586–9. 10.1097/mao.0000000000000222 24232060

[5] Juan ID Cordero CJR Carmenates IP. Sudden sensorineural hearing loss: a review. J Otolaryngol ENT Res. (2024) 16:47–9. 10.15406/joentr.2024.16.00548

[6] Pitaro J Bechor-Fellner A Gavriel H Marom T Eviatar E. Sudden sensorineural hearing loss in children: etiology, management, and outcome. Int J Pediatr Otorhinolaryngol. (2016) 82:34–7. 10.1016/j.ijporl.2015.12.022 26857312

[7] Reading JCS Hall A Nash R. Paediatric sudden sensorineural hearing loss. Pooled analysis and systematic review. J Int Adv Otol. (2021) 17:64–71. 10.5152/iao.2020.8902 33605224 PMC7901427

[8] Chen K Jiang H Zong L Wu X. Side-related differences in sudden sensorineural hearing loss in children. Int J Pediatr Otorhinolaryngol. (2018) 114:5–8. 10.1016/j.ijporl.2018.08.022 30262366

[9] Wood JW Shaffer AD Kitsko D Chi DH. Sudden sensorineural hearing loss in children-management and outcomes: a meta-analysis. Laryngoscope. (2021) 131:425–34. 10.1002/lary.28829 32673420

[10] Balduzzi S Rücker G Schwarzer G. How to perform a meta-analysis with R: a practical tutorial. Evid Based Ment Health. (2019) 22:153–60. 10.1136/ebmental-2019-300117 31563865 PMC10231495

[11] Slim K Nini E Forestier D Kwiatkowski F Panis Y Chipponi J. Methodological index for non-randomized studies (minors): development and validation of a new instrument. ANZ J Surg. (2003) 73:712–6. 10.1046/j.1445-2197.2003.02748.x 12956787

[12] Khan W Khan M Alradwan H Williams R Simunovic N Ayeni OR. Utility of intra-articular hip injections for femoroacetabular impingement: a systematic review. Orthop J Sports Med. (2015) 3:2325967115601030. 10.1177/2325967115601030 26535395 PMC4622294

[13] Moher D Liberati A Tetzlaff J Altman DG. Preferred reporting items for systematic reviews and meta-analyses: the PRISMA Statement. Open Med. (2009) 3:e123–130. 10.1093/ptj/89.9.873 21603045 PMC3090117

[14] Ao M Deng J Qi X He G. [Clinical comparison of idiopathic sudden deafness in children and the elderly]. J Clin Otorhinolaryngol Head Neck Surg. (2015) 29:1279–83. 10.13201/j.issn.1001-1781.2015.14.013 26672243

[15] Li F Xue X Wang L Yang F Wang H Guan J. [Prognostic factors of sudden sensorineural hearing loss in children]. J Clin Otorhinolaryngol Head Neck Surg. (2015) 29:1931–5. 10.13201/j.issn.1001-1781.2015.22.001 26911052

[16] Qian Y Zhong S Hu G Kang H Wang L Lei Y. Sudden sensorineural hearing loss in children: a report of 75 cases. Otol Neurotol. (2018) 39:1018–24. 10.1097/mao.0000000000001891 30063499 PMC6104722

[17] Xu J Fu Y. [Prognosis of 109 cases of sudden sensorineural hearing loss in children]. Lin Chung Er Bi Yan Hou Tou Jing Wai Ke Za Zhi. (2024) 38:598–603. 10.13201/j.issn.2096-7993.2024.07.008 PMC11599957 38973038

[18] Chung JH Cho SH Jeong JH Park CW Lee SH. Multivariate analysis of prognostic factors for idiopathic sudden sensorineural hearing loss in children. Laryngoscope. (2015) 125:2209–15. 10.1002/lary.25196 25689850

[19] Ha R Lim BW Kim DH Park JW Cho CH Lee JH. Predictive values of neutrophil to lymphocyte ratio (NLR), platelet to lymphocyte ratio (PLR), and other prognostic factors in pediatric idiopathic sudden sensorineural hearing loss. Int J Pediatr Otorhinolaryngol. (2019) 120:134–9. 10.1016/j.ijporl.2019.02.023 30784810

[20] Kim JY Han JJ Sunwoo WS Koo JW Oh SH Park MH Sudden sensorineural hearing loss in children and adolescents: clinical characteristics and age-related prognosis. Auris Nasus Larynx. (2018) 45:447–55. 10.1016/j.anl.2017.08.010 28888426

[21] Xiao L Liang J Li X Du X Yao H Ding L Analysis of clinical features and prognostic correlation factors of sudden sensorineural hearing loss in children. Int J Pediatr Otorhinolaryngol. (2023) 164:111400. 10.1016/j.ijporl.2022.111400 36446225

[22] Li FJ Wang DY Wang HY Wang L Yang FB Lan L Clinical study on 136 children with sudden sensorineural hearing loss. Chin Med J (Engl). (2016) 129:946–52. 10.4103/0366-6999.179791 27064040 PMC4831530

[23] Kizilay A Koca ÇF. Pediatric sudden sensorineural hearing loss. J Craniofac Surg. (2016) 27:e364–6. 10.1097/scs.0000000000002630 27171971

[24] Hung WC Lin KY Cheng PW Young YH. Sudden deafness: a comparison between age groups. Int J Audiol. (2021) 60:911–6. 10.1080/14992027.2021.1900611 33752575

[25] Qian Y. *A clinical study on the treatment and prognosis of sudden sensorineural hearing loss, association of SIK3 polymorphism with sudden sensorineural hearing loss in Han Chinese*. [dissertation thesis]. Chongqing Medical University, Chongqing (2019).

[26] Lu Y Zhou L Imrit TS Liu A. Sudden sensorineural hearing loss in children: clinical characteristics, etiology, treatment outcomes, and prognostic factors. Otol Neurotol. (2019) 40:446–53. 10.1097/mao.0000000000002190 30870353 PMC6426351

[27] Lee JS Hong SK Kim DH Lee JH Lee HJ Park B The neutrophil-to-lymphocyte ratio in children with sudden sensorineural hearing loss: a retrospective study. Acta Otolaryngol. (2017) 137:35–8. 10.1080/00016489.2016.1217561 27598228

[28] Mattox DE Simmons FB. Natural history of sudden sensorineural hearing loss. Ann Otol Rhinol Laryngol. (1977) 86:463–80. 10.1177/000348947708600406 889223

[29] Ashtiani MK Firouzi F Bastaninejad S Dabiri S Nasirmohtaram S Saeedi N Efficacy of systemic and intratympanic corticosteroid combination therapy versus intratympanic or systemic therapy in patients with idiopathic sudden sensorineural hearing loss: a randomized controlled trial. Eur Arch Otorhinolaryngol. (2018) 275:89–97. 10.1007/s00405-017-4808-0 29149379

[30] Bulğurcu S Dikilitaş B Arslan İB Çukurova İ. Neutrophil-to-lymphocyte and platelet-to-lymphocyte ratios in pediatric patients with idiopathic sudden hearing loss. J Int Adv Otol. (2017) 13:217–20. 10.5152/iao.2017.3404 28414280

[31] Chandrasekhar SS Tsai Do BS Schwartz SR Bontempo LJ Faucett EA Finestone SA Clinical practice guideline: sudden hearing loss (update) executive summary. Otolaryngol Head Neck Surg. (2019) 161:195–210. 10.1177/0194599819859883 31369349

[32] Editorial Board of Chinese Journal of Otorhinolaryngology Head and Neck Surgery, Society of Otorhinolaryngology Head and Neck Surgery, CMA. Guidelines for the diagnosis and treatment of sudden sensorineural hearing loss. Chin J Otorhinolaryngol Head Neck Surg. (2015) 50:443–7. 10.3760/cma.j.issn.1673-0860.2015.06.002

[33] Marx M Younes E Chandrasekhar SS Ito J Plontke S O'Leary S International consensus (ICON) on treatment of sudden sensorineural hearing loss. Eur Ann Otorhinolaryngol Head Neck Dis. (2018) 135:S23–8. 10.1016/j.anorl.2017.12.011 29396226

[34] Lee HS Lee YJ Kang BS Lee BD Lee JS. A clinical analysis of sudden sensorineural hearing loss cases. Korean J Audiol. (2014) 18:69–75. 10.7874/kja.2014.18.2.69 25279228 PMC4181056

[35] Gonzalez-Garcia M Prieto-Sanchez-Puerta L Dominguez-Duran E Sanchez-Gomez S. Auditory prognosis of patients with sudden sensorineural hearing loss in relation to the presence of acute vestibular syndrome: a systematic literature review and meta-analysis. Ear Hear. (2025) 46:8–15. 10.1097/aud.0000000000001576 39252156

[36] Byun H Chung JH Lee SH. Clinical implications of posterior semicircular canal function in idiopathic sudden sensorineural hearing loss. Sci Rep. (2020) 10:8313. 10.1038/s41598-020-65294-5 32433568 PMC7239936

[37] Seo HW Chung J Byun H Lee SH. Vestibular mapping assessment in idiopathic sudden sensorineural hearing loss. Ear Hear. (2021) 43:242–9. 10.1097/aud.0000000000001129 34524151

[38] Stachler RJ Chandrasekhar SS Archer SM Rosenfeld RM Schwartz SR Barrs DM. Clinical practice guideline: sudden hearing loss. Otolaryngol Head Neck Surg. (2012) 146:S1–35. 10.1177/0194599812436449 22383545

[39] Liu YX Yu HQ Li HW. Interpretation of guidelines/statements/consensus on idiopathic sudden sensorineural hearing loss. Chin J Otorhinolaryngol Head Neck Surg. (2023) 58(6):637–42. 10.3760/cma.j.cn115330-20221020-00619 37339908

